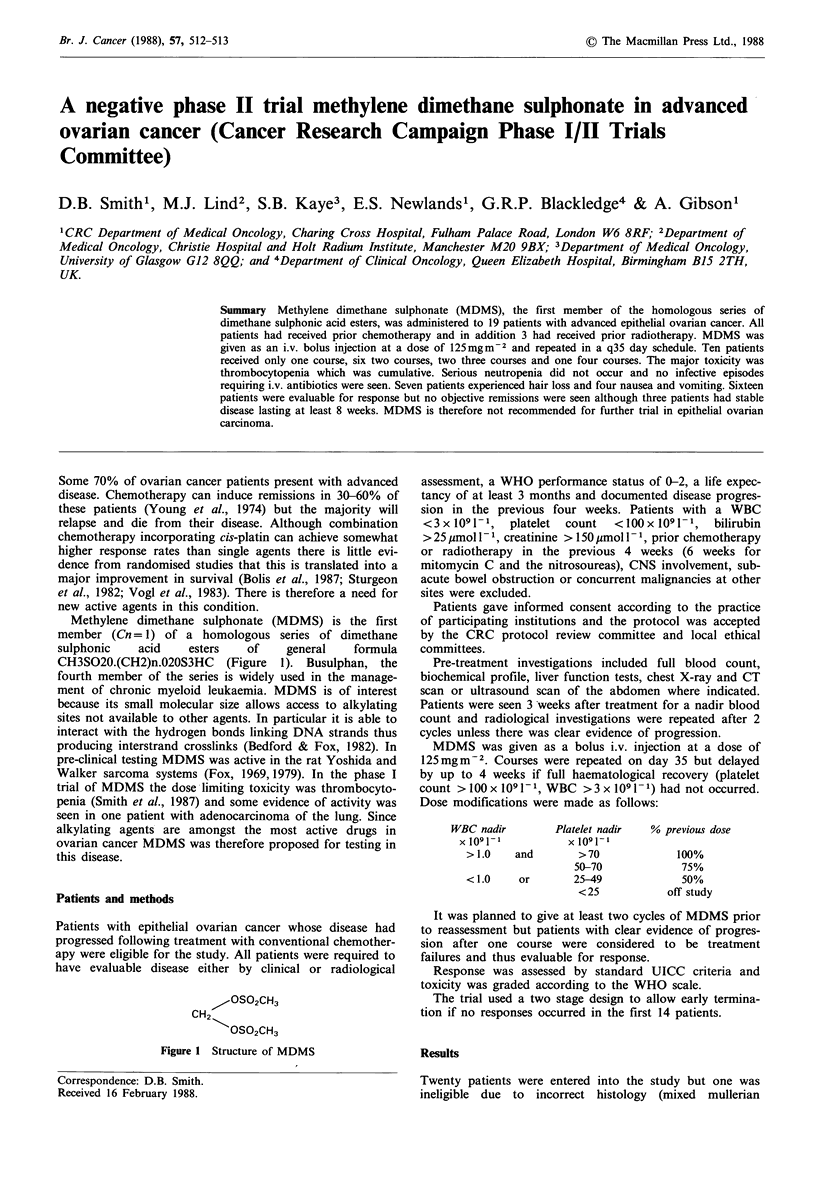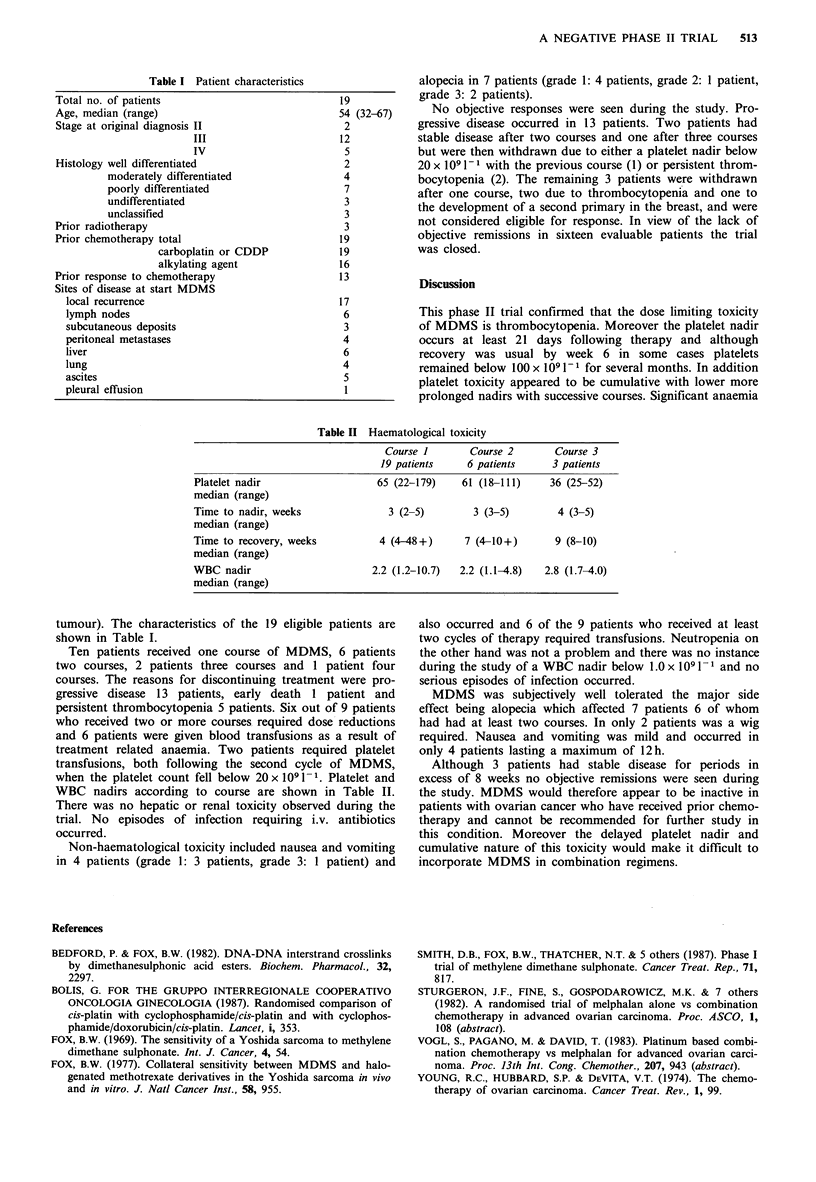# A negative phase II trial methylene dimethane sulphonate in advanced ovarian cancer (Cancer Research Campaign Phase I/II Trials Committee).

**DOI:** 10.1038/bjc.1988.116

**Published:** 1988-05

**Authors:** D. B. Smith, M. J. Lind, S. B. Kaye, E. S. Newlands, G. R. Blackledge, A. Gibson

**Affiliations:** CRC Department of Medical Oncology, Charing Cross Hospital, London, UK.

## Abstract

Methylene dimethane sulphonate (MDMS), the first member of the homologous series of dimethane sulphonic acid esters, was administered to 19 patients with advanced epithelial ovarian cancer. All patients had received prior chemotherapy and in addition 3 had received prior radiotherapy. MDMS was given as an i.v. bolus injection at a dose of 125mg m-2 and repeated in a q35 day schedule. Ten patients received only one course, six two courses, two three courses and one four courses. The major toxicity was thrombocytopenia which was cumulative. Serious neutropenia did not occur and no infective episodes requiring i.v. antibiotics were seen. Seven patients experience hair loss and four nausea and vomiting. Sixteen patients were evaluable for response but no objective remissions were seen although three patients had stable disease lasting at least 8 weeks. MDMS is therefore not recommended for further trial in epithelial ovarian carcinoma.


*Br. J. Cancer* (1988), **57**, 512–513 © The Macmillan Press Ltd., 1988

### A negative phase II trial methylene dimethane sulphonate in advanced ovarian cancer (Cancer Research Campaign Phase I/II Trials Committee)

D.B. Smith[1], M.J. Lind[2], S.B. Kaye[3], E.S. Newlands[1], G.R.P. Blackledge[4] & A. Gibson[1]

[1]*CRC Department of Medical Oncology, Charing Cross Hospital, Fulham Palace Road, London W6 8RF;* [2]*Department of Medical Oncology, Christie Hospital and Holt Radium Institute, Manchester M20 9BX;* [3]*Department of Medical Oncology, University of Glasgow G12 8QQ; and* [4]*Department of Clinical Oncology, Queen Elizabeth Hospital, Birmingham B15 2TH, UK.*

**Summary** Methylene dimethane sulphonate (MDMS), the first member of the homologous series of dimethane sulphonic acid esters, was administered to 19 patients with advanced epithelial ovarian cancer. All patients had received prior chemotherapy and in addition 3 had received prior radiotherapy. MDMS was given as an i.v. bolus injection at a dose of $125 \mathrm{mg\,m^{-2}}$ and repeated in a q35 day schedule. Ten patients received only one course, six two courses, two three courses and one four courses. The major toxicity was thrombocytopenia which was cumulative. Serious neutropenia did not occur and no infective episodes requiring i.v. antibiotics were seen. Seven patients experienced hair loss and four nausea and vomiting. Sixteen patients were evaluable for response but no objective remissions were seen although three patients had stable disease lasting at least 8 weeks. MDMS is therefore not recommended for further trial in epithelial ovarian carcinoma.

Some 70% of ovarian cancer patients present with advanced disease. Chemotherapy can induce remissions in 30–60% of these patients (Young *et al.*, 1974) but the majority will relapse and die from their disease. Although combination chemotherapy incorporating *cis*-platin can achieve somewhat higher response rates than single agents there is little evidence from randomised studies that this is translated into a major improvement in survival (Bolis *et al.*, 1987; Sturgeon *et al.*, 1982; Vogl *et al.*, 1983). There is therefore a need for new active agents in this condition.

Methylene dimethane sulphonate (MDMS) is the first member ($Cn = 1$) of a homologous series of dimethane sulphonic acid esters of general formula CH3SO20.(CH2)n.020S3HC (Figure 1). Busulphan, the fourth member of the series is widely used in the management of chronic myeloid leukaemia. MDMS is of interest because its small molecular size allows access to alkylating sites not available to other agents. In particular it is able to interact with the hydrogen bonds linking DNA strands thus producing interstrand crosslinks (Bedford & Fox, 1982). In pre-clinical testing MDMS was active in the rat Yoshida and Walker sarcoma systems (Fox, 1969, 1979). In the phase I trial of MDMS the dose limiting toxicity was thrombocytopenia (Smith *et al.*, 1987) and some evidence of activity was seen in one patient with adenocarcinoma of the lung. Since alkylating agents are amongst the most active drugs in ovarian cancer MDMS was therefore proposed for testing in this disease.

### Patients and methods

Patients with epithelial ovarian cancer whose disease had progressed following treatment with conventional chemotherapy were eligible for the study. All patients were required to have evaluable disease either by clinical or radiological

**Figure 1** Structure of MDMS

Correspondence: D.B. Smith.
Received 16 February 1988.

assessment, a WHO performance status of 0–2, a life expectancy of at least 3 months and documented disease progression in the previous four weeks. Patients with a WBC $<3\times10^{9}\,\mathrm{l^{-1}}$, platelet count $<100\times10^{9}\,\mathrm{l^{-1}}$, bilirubin $>25\,\mu\mathrm{mol\,l^{-1}}$, creatinine $>150\,\mu\mathrm{mol\,l^{-1}}$, prior chemotherapy or radiotherapy in the previous 4 weeks (6 weeks for mitomycin C and the nitrosoureas), CNS involvement, subacute bowel obstruction or concurrent malignancies at other sites were excluded.

Patients gave informed consent according to the practice of participating institutions and the protocol was accepted by the CRC protocol review committee and local ethical committees.

Pre-treatment investigations included full blood count, biochemical profile, liver function tests, chest X-ray and CT scan or ultrasound scan of the abdomen where indicated. Patients were seen 3 weeks after treatment for a nadir blood count and radiological investigations were repeated after 2 cycles unless there was clear evidence of progression.

MDMS was given as a bolus i.v. injection at a dose of $125\,\mathrm{mg\,m^{-2}}$. Courses were repeated on day 35 but delayed by up to 4 weeks if full haematological recovery (platelet count $>100\times10^{9}\,\mathrm{l^{-1}}$, WBC $>3\times10^{9}\,\mathrm{l^{-1}}$) had not occurred. Dose modifications were made as follows:

| *WBC nadir* $\times10^{9}\,\mathrm{l^{-1}}$ | | *Platelet nadir* $\times10^{9}\,\mathrm{l^{-1}}$ | *% previous dose* |
|---|---|---|---|
| >1.0 | and | >70 | 100% |
| | | 50–70 | 75% |
| <1.0 | or | 25–49 | 50% |
| | | <25 | off study |

It was planned to give at least two cycles of MDMS prior to reassessment but patients with clear evidence of progression after one course were considered to be treatment failures and thus evaluable for response.

Response was assessed by standard UICC criteria and toxicity was graded according to the WHO scale.

The trial used a two stage design to allow early termination if no responses occurred in the first 14 patients.

### Results

Twenty patients were entered into the study but one was ineligible due to incorrect histology (mixed mullerian

**Table I** Patient characteristics

| | |
|---|---|
| Total no. of patients | 19 |
| Age, median (range) | 54 (32–67) |
| Stage at original diagnosis II | 2 |
| III | 12 |
| IV | 5 |
| Histology well differentiated | 2 |
| moderately differentiated | 4 |
| poorly differentiated | 7 |
| undifferentiated | 3 |
| unclassified | 3 |
| Prior radiotherapy | 3 |
| Prior chemotherapy total | 19 |
| carboplatin or CDDP | 19 |
| alkylating agent | 16 |
| Prior response to chemotherapy | 13 |
| Sites of disease at start MDMS | |
| local recurrence | 17 |
| lymph nodes | 6 |
| subcutaneous deposits | 3 |
| peritoneal metastases | 4 |
| liver | 6 |
| lung | 4 |
| ascites | 5 |
| pleural effusion | 1 |

**Table II** Haematological toxicity

| | *Course 1* *19 patients* | *Course 2* *6 patients* | *Course 3* *3 patients* |
|---|---|---|---|
| Platelet nadir median (range) | 65 (22–179) | 61 (18–111) | 36 (25–52) |
| Time to nadir, weeks median (range) | 3 (2–5) | 3 (3–5) | 4 (3–5) |
| Time to recovery, weeks median (range) | 4 (4–48+) | 7 (4–10+) | 9 (8–10) |
| WBC nadir median (range) | 2.2 (1.2–10.7) | 2.2 (1.1–4.8) | 2.8 (1.7–4.0) |

tumour). The characteristics of the 19 eligible patients are shown in Table I.

Ten patients received one course of MDMS, 6 patients two courses, 2 patients three courses and 1 patient four courses. The reasons for discontinuing treatment were progressive disease 13 patients, early death 1 patient and persistent thrombocytopenia 5 patients. Six out of 9 patients who received two or more courses required dose reductions and 6 patients were given blood transfusions as a result of treatment related anaemia. Two patients required platelet transfusions, both following the second cycle of MDMS, when the platelet count fell below $20 \times 10^9 l^{-1}$. Platelet and WBC nadirs according to course are shown in Table II. There was no hepatic or renal toxicity observed during the trial. No episodes of infection requiring i.v. antibiotics occurred.

Non-haematological toxicity included nausea and vomiting in 4 patients (grade 1: 3 patients, grade 3: 1 patient) and alopecia in 7 patients (grade 1: 4 patients, grade 2: 1 patient, grade 3: 2 patients).

No objective responses were seen during the study. Progressive disease occurred in 13 patients. Two patients had stable disease after two courses and one after three courses but were then withdrawn due to either a platelet nadir below $20 \times 10^9 l^{-1}$ with the previous course (1) or persistent thrombocytopenia (2). The remaining 3 patients were withdrawn after one course, two due to thrombocytopenia and one to the development of a second primary in the breast, and were not considered eligible for response. In view of the lack of objective remissions in sixteen evaluable patients the trial was closed.

### Discussion

This phase II trial confirmed that the dose limiting toxicity of MDMS is thrombocytopenia. Moreover the platelet nadir occurs at least 21 days following therapy and although recovery was usual by week 6 in some cases platelets remained below $100 \times 10^9 l^{-1}$ for several months. In addition platelet toxicity appeared to be cumulative with lower more prolonged nadirs with successive courses. Significant anaemia also occurred and 6 of the 9 patients who received at least two cycles of therapy required transfusions. Neutropenia on the other hand was not a problem and there was no instance during the study of a WBC nadir below $1.0 \times 10^9 l^{-1}$ and no serious episodes of infection occurred.

MDMS was subjectively well tolerated the major side effect being alopecia which affected 7 patients 6 of whom had had at least two courses. In only 2 patients was a wig required. Nausea and vomiting was mild and occurred in only 4 patients lasting a maximum of 12 h.

Although 3 patients had stable disease for periods in excess of 8 weeks no objective remissions were seen during the study. MDMS would therefore appear to be inactive in patients with ovarian cancer who have received prior chemotherapy and cannot be recommended for further study in this condition. Moreover the delayed platelet nadir and cumulative nature of this toxicity would make it difficult to incorporate MDMS in combination regimens.

### References

BEDFORD, P. & FOX, B.W. (1982). DNA-DNA interstrand crosslinks by dimethanesulphonic acid esters. *Biochem. Pharmacol.*, **32**, 2297.

BOLIS, G. FOR THE GRUPPO INTERREGIONALE COOPERATIVO ONCOLOGIA GINECOLOGIA (1987). Randomised comparison of *cis*-platin with cyclophosphamide/*cis*-platin and with cyclophosphamide/doxorubicin/*cis*-platin. *Lancet*, **i**, 353.

FOX, B.W. (1969). The sensitivity of a Yoshida sarcoma to methylene dimethane sulphonate. *Int. J. Cancer*, **4**, 54.

FOX, B.W. (1977). Collateral sensitivity between MDMS and halogenated methotrexate derivatives in the Yoshida sarcoma *in vivo* and *in vitro*. *J. Natl Cancer Inst.*, **58**, 955.

SMITH, D.B., FOX, B.W., THATCHER, N.T. & 5 others (1987). Phase I trial of methylene dimethane sulphonate. *Cancer Treat. Rep.*, **71**, 817.

STURGERON, J.F., FINE, S., GOSPODAROWICZ, M.K. & 7 others (1982). A randomised trial of melphalan alone vs combination chemotherapy in advanced ovarian carcinoma. *Proc. ASCO*, **1**, 108 (*abstract*).

VOGL, S., PAGANO, M. & DAVID, T. (1983). Platinum based combination chemotherapy vs melphalan for advanced ovarian carcinoma. *Proc. 13th Int. Cong. Chemother.*, **207**, 943 (*abstract*).

YOUNG, R.C., HUBBARD, S.P. & DEVITA, V.T. (1974). The chemotherapy of ovarian carcinoma. *Cancer Treat. Rev.*, **1**, 99.